# Behavioral evidence of impaired self-referential processing in patients with affective disorders and first-episode schizophrenia

**DOI:** 10.1038/s41598-024-60498-5

**Published:** 2024-05-10

**Authors:** Yanli Zhao, Jiahua Xu, Jiangyue Hong, Xuejing Xu, Hongzhen Fan, Jinguo Zhang, Dong Li, Jingxu Chen, Yaxue Wu, Yanli Li, Yunlong Tan, Shuping Tan

**Affiliations:** 1 Beijing HuiLongGuan Hospital, Peking University HuiLongGuan Clinical Medical School, Beijing, 100096 People’s Republic of China; 2 Temple University, Philadelphia, PA 19122 USA

**Keywords:** Self-reference effect, Schizophrenia, Bipolar disorder, Major depressive disorder, Self-referential processing, Mother-referential processing, Bipolar disorder, Depression, Schizophrenia, Human behaviour, Cognitive neuroscience, Social neuroscience

## Abstract

Despite the critical role of self-disturbance in psychiatric diagnosis and treatment, its diverse behavioral manifestations remain poorly understood. This investigation aimed to elucidate unique patterns of self-referential processing in affective disorders and first-episode schizophrenia. A total of 156 participants (41 first-episode schizophrenia [SZ], 33 bipolar disorder [BD], 44 major depressive disorder [MDD], and 38 healthy controls [HC]) engaged in a self-referential effect (SRE) task, assessing trait adjectives for self-descriptiveness, applicability to mother, or others, followed by an unexpected recognition test. All groups displayed preferential self- and mother-referential processing with no significant differences in recognition scores. However, MDD patients showed significantly enhanced self-referential recognition scores and increased bias compared to HC, first-episode SZ, and BD. The present study provides empirical evidence for increased self-focus in MDD and demonstrates that first-episode SZ and BD patients maintain intact self-referential processing abilities. These findings refine our understanding of self-referential processing impairments across psychiatric conditions, suggesting that it could serve as a supplementary measure for assessing treatment response in first-episode SZ and potentially function as a discriminative diagnostic criterion between MDD and BD.

## Introduction

Self-disturbance constitutes a salient transdiagnostic feature across a wide array of mental health conditions. The concept of the self is multifaceted, as posited by various theoretical frameworks^1,2^. Among its diverse dimensions, two central aspects are the pre-reflective and reflective selves^1^. The pre-reflective or minimal self facilitates an immediate sense of being the experiencer, existing in the present moment without temporal extension, while the reflective or narrative self encompasses the totality of one's identity, enabling comprehension of oneself within the continuum of time^3^.

In the present study, we specifically direct our attention to the reflective self dimension, operationalized through the self-reference effect (SRE) paradigm^4^ in patients diagnosed with affective disorders and schizophrenia. The SRE task typically consists of dual stages: encoding and recognition. During the encoding stage, participants are tasked with judging whether a series of trait adjectives apply to themselves or another individual, thus engaging both self-reflection (often referred to as self-referential processing) and reflection on others (referred to as other-referential processing). Subsequently, an unanticipated recognition phase follows after a brief interval, where participants must discern which words were previously encountered during the encoding session. Empirical evidence from studies involving healthy adult populations has consistently demonstrated that recognition performance is significantly enhanced when items have been encoded under self-referential conditions relative to other-referential contexts, a phenomenon widely recognized as the SRE^5^. The manifestation of the SRE critically depends upon an individual's intact capacity for self-referential processing, i.e., the conscious cognitive appraisal of information relevant to the self^6,7^. Therefore, any impairment in the SRE can serve as a reliable behavioral marker indicative of compromised self-referential processing functionality.

Self-referential processing constitutes a fundamental aspect of social cognition and is pivotal for social adaptation^8,9^. Its impairment is pertinent to the manifestation of psychosis, maladaptive social functioning, and diminished illness awareness in schizophrenia (SZ)^10,11^. Major Depressive Disorder (MDD) is marked by an intensified self-focused cognitive pattern, i.e., the tendency for individuals to engage in a persistent and repetitive form of self-referential processing, frequently accompanied by a self-critical perspective and the growing empirical evidence supports the critical involvement of self-referential processing mechanisms across various stages of MDD^12^. Consequently, the study of self-referential processing in mental health disorders has attracted substantial interest from numerous scholars. Previous research on self-referential processing in affective disorders and schizophrenia has largely employed functional magnetic resonance imaging (fMRI) or event-related potential (ERP) methodologies to directly probe this process, offering neurobiological evidence for the anomalies observed in self-referential processing within these conditions^13–18^. Despite their value, these techniques are often time-consuming and demand higher participant compliance compared to behavioral tasks, which offer a more straightforward and expedient experimental avenue. However, there is currently a scarcity of behavioral research specifically addressing the SRE in psychiatric populations. Findings from prior studies^19^ have indicated abolished SRE and compromised self-referential processing in SZ patients. Conversely, certain investigations have shown that first-episode SZ patients maintain intact SRE and self-referential processing abilities but exhibit deficits in mother-referential (intimate other) information processing when compared to healthy controls (HC)^20^. A small-scale study on bipolar disorder (BD) has revealed that impairments in self-referential and other-referential processing may be exclusive to BD patients with comorbid psychotic symptoms, whereas those without accompanying psychotic features exhibit intact self-referential processing capabilities^18^. More recent work has revealed an amplified self-referential advantage in MDD patients over HC, suggesting excessive self-focus in MDD^21^, although some studies do not report such augmentation^17^.

In summary, the current body of behavioral research exploring self-referential processing in schizophrenia and affective disorders is relatively sparse and exhibits inconsistencies. To our knowledge, few studies have systematically investigated the presence and nature of self-referential processing impairments in affective disorder and first-episode SZ patients using a common behavioral task. This lack of comparative data is crucial for enhancing diagnostic precision, differential diagnosis, and the design of efficacious therapeutic interventions in mental health disorders. Notably, previous literature suggests that the Chinese self-concept embodies an interdependent self where the self includes the mother^22^. Therefore, for a comprehensive understanding of self-disturbances in Chinese cultural contexts, it is essential to consider mother-referential processing. Addressing these gaps, the present study adopted an established SRE task^20^ to explore whether patients with affective disorders and first-episode SZ exhibit impairments in interdependent self-representation, i.e., dysfunctional self- and/or mother-referential processing. Given that self-referential processing abnormalities in BD may be influenced by the presence or absence of psychotic symptoms^18^ and the phase of the disorder (depressive or manic episode), we refrained from formulating specific hypotheses regarding BD outcomes. We anticipated that MDD patients would display better recognition scores in self- and/or mother-referential processing due to heightened self-focus^12^. Furthermore, we hypothesized that first-episode SZ patients would reveal dysfunction in intimate other-referential processing^20^.

## Results

### Encoding phase

**Figure 1:**
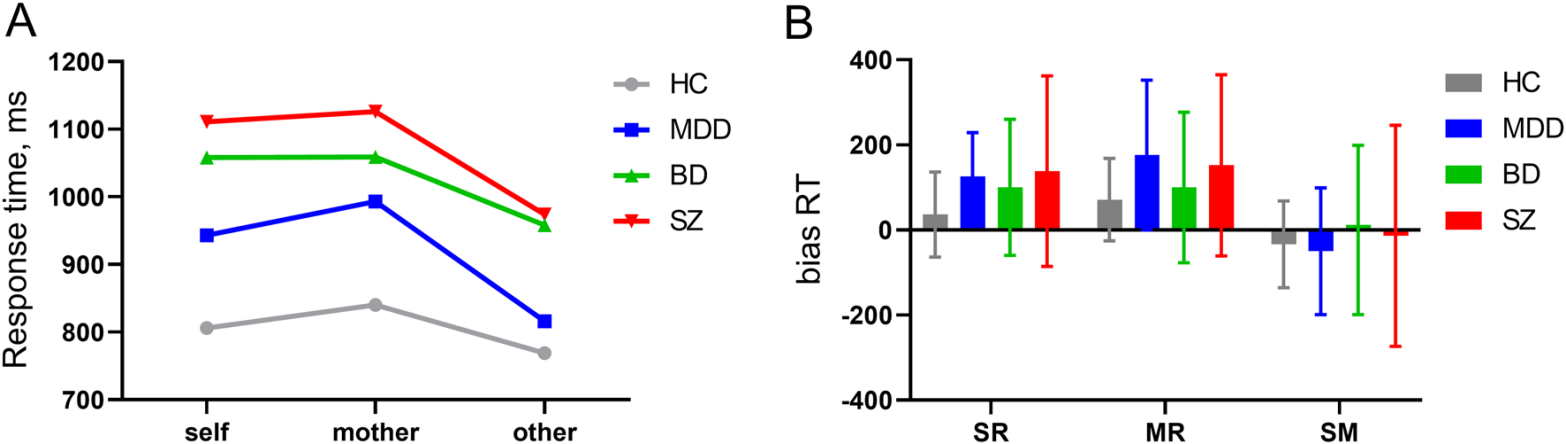
The behavioral performance during encoding phase. (**A**) The line graphs depict the response time (RT) among patients with first-episode schizophrenia (SZ), bipolar disorder (BD), major depressive disorder (MDD) and healthy controls (HC). In the context of mother-referential processing, RTs were longer for patients with MDD compared to HC. Under all three experiment conditions, both first-episode SZ and BD patients exhibited prolonged RTs relative to HC. Within self-referential processing conditions, MDD patients showed shorter RTs than first-episode SZ patients. When it came to other-referential processing, MDD patients demonstrated shorter RTs compared to first-episode SZ and BD patients. In patient cohorts, there is no discernible difference in RTs between the self-referential and mother-referential processing conditions; however, both consistently exhibit longer RTs than those recorded under the other-referential condition. In contrast, among the control group, a clear distinction exists such that RTs are significantly longer under the mother-referential condition relative to the other-referential condition alone. (**B**) The bar graphs depict the group differences of bias RT among first-episode SZ, BD, MDD and HC. The normal group had lower bias RT compared to first-episode SZ patients and MDD patients. **p < 0.01; *p < 0.05. SM: self-mother, MR: Mother-other, SR: self-other.

The results of the 4 (group:HC/MDD/BD/first-episode SZ) × 3 (condition: self/mother/other) repeated measures ANOVA on RT revealed a significant interaction between condition and group [*F* (6286) = 2.30, *P* = 0.035; Table 1; Fig. 1A]. Simple effects analyses disclosed that in the HC group, participants exhibited significantly longer RT during other-referential processing compared to mother-referential processing, *P* = 0.035; however, there was no significant difference between self-referential and other-referential processing, *P* = 0.471. Moreover, no significant difference in RT was observed between self- and mother-referential processing, *P* = 0.777. For MDD, BD, and first-episode SZ groups, RT were significantly longer during other-referential processing than during both self-referential and mother-referential processing (all *P* < 0.01). In contrast, no significant differences in RT were detected between self- and mother-referential processing within these clinical groups (all *P* > 0.1).

**Table 1:** Encoding RT data for HC and patients with MDD, BD, and first-episode SZ.

Task	SZ (n = 41)	BD (n = 33)	MDD (n = 35)	HC (n = 38)
RT (ms)	Mean ± SD	Mean ± SD	Mean ± SD	Mean ± SD
Self-referential	1111 ± 343	1058 ± 269	943 ± 171	806 ± 136
Other-referential	974 ± 203	958 ± 231	816 ± 140	769 ± 138
Mother-referential	1126 ± 290	1059 ± 296	993 ± 250	840 ± 167
SR bias RT	138 ± 224	100 ± 160	126 ± 103	36 ± 100
MR bias RT	152 ± 213	100 ± 177	176 ± 176	71 ± 97
SM bias RT	− 14 ± 260	− .14 ± 199	− 50 ± 149	− 34 ± 102

SZ: first-episode schizophrenia; BD: bipolar disorder; MDD: major depressive disorder; HC: healthy controls; SR bias RT, MR bias RT and the SM bias RT were respectively defined as the response time between self-and other-referential conditions, the differential response time between mother-and other-referential conditions, and the differential response time between self-and mother-referential conditions.

In the context of self-referential processing, while there was no significant difference in RT between the HC and MDD groups (*P* = 0.115), the controls had significantly shorter RT compared to those with BD and first-episode SZ (both *P* < 0.001). Additionally, the RT for the MDD group were significantly lower than those for the first-episode SZ group (*P* = 0.021); notably, no significant difference was found between the BD and first-episode SZ groups (*P* = 1.000). Under mother-referential processing conditions, the HC group displayed shorter RT than those in the MDD (*P* = 0.071), BD (*P* = 0.003), and first-episode schizophrenic groups (*P* < 0.001); however, among the three patient groups, no significant differences in RT were observed (all *P* > 0.1). Regarding other-referential processing, no significant difference in RT was detected between the HC and MDD groups (*P* = 1.000); however, the control group showed significantly shorter RT when compared to BD and first-episode schizophrenic groups (both *P* < 0.001). Furthermore, the RT for the MDD group were also significantly shorter than those for the BD and first-episode SZ groups (both *P* < 0.05). As before, no significant difference in RT emerged between the BD and first-episode SZ groups for other-referential processing (*P* = 1.000).

The results of the repeated-measures ANOVA on bias RT revealed a significant main effect of condition [*F* (2286) = 33.08, *P* < 0.001]. Further pairwise comparisons disclosed that the SM bias RT values were significantly shorter than those of SR bias and MR bias (*P*-values for both comparisons were less than 0.001); however, no significant difference was found between SR bias and MR bias, *P* = 0.341. Moreover, a significant main effect of group was observed [*F* (3, 143) = 3.17, *P* = 0.026; Table 1; Fig. 1B]. Subsequent post-hoc tests indicated that the normal group had significantly lower bias RT compared to first-episode SZ patients (*P* = 0.029) and MDD patients (marginally significant at *P* = 0.099). For all other pair-wise comparisons among groups, no statistically significant differences were detected with p-values being greater than or equal to 0.547.

### Recognition phase

**Figure 2:**
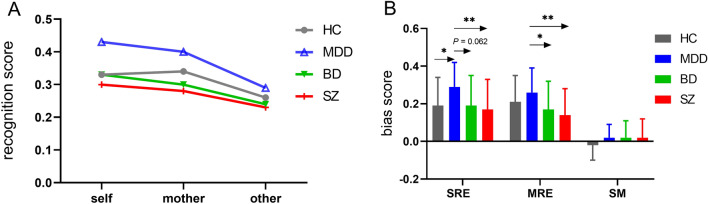
The behavioral performance during recognition phase. (**A**) The line graphs depict the recognition score among patients with first-episode schizophrenia (SZ), bipolar disorder (BD), major depressive disorder (MDD), and healthy controls (HC). All participants, including HC and patients with MDD, BD, and first-episode SZ, displayed both self-referential and mother-referential bias in recognition memory, characterized by a pattern where scores for self- and mother-referenced items were higher than those for other-referenced items (self-recognition = mother-recognition > other-recognition). Additionally, participants with MDD exhibited significantly elevated self-referential recognition scores compared to HC, first-episode SZ, and BD patients. While MDD patients outperformed first-episode SZ and BD patients in recognizing information related to their mothers, no statistically discernible difference was found when comparing MDD patients to HC. (**B**) The bar graphs depict the group differences of bias score among first-episode SZ, BD, MDD, and HC. Patients with MDD exhibit higher SRE bias scores compared to those with first-episode SZ, BD, and HC. Meanwhile, Patients with MDD exhibit higher MRE bias scores compared to those with first-episode SZ and BD. **p < 0.01; *p < 0.05. SM: self-mother, MRE: Mother-referential effect, SRE: self-referential effect.

The results of a 4 (group: HC/MDD/BD/first-episode SZ) × 3 (condition: self/mother/other) repeated measures ANOVA on recognition scores revealed a significant interaction between condition and group [*F* (6, 286) = 3.06, *P* = 0.006; Table 2; Fig. 2A]. The simple effect analysis indicated that the recognition scores in all participants revealed significant difference across the three conditions: self-referential = mother-referential (pairwise *P* ≥ 0.257) > other-referential (pairwise *P* ≤ 0.004).

**Table2:** Recognition score and bias score for HC and patients with affective disorders and first-episode SZ.

Task	SZ(n = 41)	BD(n = 33)	MDD(n = 35)	HC(n = 38)
Recognition score	Mean ± SD	Mean ± SD	Mean ± SD	Mean ± SD
Self-referential	0.30 ± 0.18	0.33 ± 0.18	0.43 ± 0.13	0.33 ± 0.14
mother-referential	0.28 ± 0.16	0.30 ± 0.16	0.40 ± 0.14	0.34 ± 0.12
other-referential	0.23 ± 0.14	0.24 ± 0.12	0.29 ± 0.12	0.26 ± 0.12
SRE bias score	0.17 ± 0.16	0.19 ± 0.16	0.29 ± 0.13	0.19 ± 0.15
MRE bias score	0.14 ± 0.14	0.17 ± 0.15	0.26 ± 0.13	0.21 ± 0.14
SM bias score	0.02 ± 0.10	0.02 ± 0.09	0.02 ± 0.07	− 0.02 ± 0.08

SZ: first-episode schizophrenia; BD: bipolar disorder; MDD: major depressive disorder; HC: healthy controls; the SRE bias score, the MRE bias score, and the SM bias score were respectively defined as the difference between the “self-referential d“ and the “other-referential d“ (“self-referential d” minus “other-referential d”), the difference between the “mother-referential d” and the “other-referential d” (“mother-referential d” minus “other-referential d“), and the difference between the “self-referential d“ and the “mother-referential d” (“self-referential d“ minus “mother-referential d“).

Moreover, it was found that MDD patients demonstrated higher self-referential recognition scores compared to first-episode SZ (*P* = 0.004), BD (*P* = 0.064), and HC participants (*P* = 0.051). Regarding maternal-referential recognition scores, MDD patients scored significantly higher than those with first-episode SZ (*P* = 0.001) and BD (*P* = 0.029), yet no significant difference was observed when compared to the HC (*P* = 0.490). Additionally, a lack of statistically discernible disparities in mother-referential processing recognition performance has been observed among individuals diagnosed with BD relative to their counterparts with first-episode SZ, and this pattern persists when contrasting each patient cohort against a matched group of HC (all [*P*-values being ≥ 0.256). No significant differences in other-referential recognition scores were detected among the four groups, with all *p*-values being ≥ 0.285.

The results of the repeated measures ANOVA on bias score revealed a significant interaction between condition and group [*F* (6286) = 4.20, *P* = 0.003; Table 2; Fig. 2B]. The results from simple effects analyses revealed that patients with MDD exhibited significantly higher self bias compared to individuals with first-episode SZ (*P* = 0.004), as well as those with BD (*P* = 0.062) and HC (*P* = 0.036). Concurrently, MDD patients also displayed a mother bias that was elevated in comparison to first-episode SZ (*P* = 0.002) and BD (*P* = 0.031) patients; However, no statistically significant difference was found when contrasting MDD patients against the normal control group (*P* = 0.453). Furthermore, for both self and mother biases, no other group comparisons reached statistical significance as all corresponding *P*-values exceeded the threshold of 0.304. Moreover, there were no discernible statistically significant differences among any two groups concerning the differential scores under self-mother referential conditions, referred to as the SM bias, with all *P*-values exceeding the minimum threshold of 0.269. Additionally, for all participants, the self bias and mother bias values were consistently higher than the SM bias values (all *p*-values < 0.001), and no statistically significant differences were observed between the self bias and mother bias scores (all *P*-values ≥ 0.257).

## Discussion

To the best of our understanding, this research pioneers the use of a unified behavioral paradigm to systematically and comparatively assess potential impairments in self-referential processing among first-episode SZ, BD, and MDD patients. The study's outcomes reveal that individuals across all three patient groups—first-episode SZ, BD, and MDD—exhibit comparable SRE and MRE patterns to those seen in HC, indicative of preserved preferential processing for information pertaining to both the self and mother domains. Furthermore, akin to HC participants, the self-conceptualization in patients also includes a mother-referential component. Notably, this investigation has revealed unique profiles of self-referential processing abilities across distinct psychiatric conditions, uncovering novel insights that deviate from previous findings in the literature.

Firstly, this study demonstrates a significantly heightened self-recognition and augmented SRE bias in MDD patients when compared to HC. However, no significant variations were observed between these groups concerning recognition performance for mother-referential or other-referential information, suggesting that MDD individuals exhibit a selectively amplified memory preference specifically for self-related content. This observation corroborates earlier behavioral research on MDD^21^ as well as prior fMRI data indicating abnormal hyperactivity within the anterior cingulate cortex during self-referential processing in MDD^12^. It is pertinent to mention that some studies have reported inconclusive results with no discernible differences in self-relevant information retrieval between acute or remitted MDD patients and HC^17^. The divergent findings might stem from the utilization of disparate behavioral instruments, particularly free recall tasks as opposed to the recognition tasks employed herein.

During the encoding phase, our analysis unveiled longer RTs among MDD patients under mother-referential conditions, coupled with increased RT biases for SR, MR, and SM relative to HC. Importantly, no significant difference was detected in RTs for tasks involving other-referential processing between the two cohorts, potentially implying an enhanced duration spent elaborating or organizing self- and mother-related information in MDD patients.

Secondly, our results indicate that there are no significant differences in any bias measures or self- and mother referential recognition between first-episode SZ patients and HC, suggesting an intact SRE and interconnected self-concept at the early stages of SZ. This contrasts with previous research^20^ that suggested preserved fundamental self-function but altered relational aspects of self in initial SZ episodes. The present study is the first to provide empirical evidence that not all individuals experiencing a first episode of schizophrenia show deficits in processing information related to intimate others, specifically mother-referential processing. Earlier studies have shown a detrimental association between recognition performance in tasks involving self- and mother-referential processing and total PANSS scores among first-episode SZ patients^20^, pointing towards a relationship where more severe symptoms are linked with poorer recognition under both self- and mother-related conditions. In this particular investigation, the symptom severity as measured by the PANSS total score for the first-episode SZ group was notably lower (51.93 ± 25.64) compared to prior literature (75.90 ± 10.41). This reduced symptomatology could potentially explain the maintenance of unimpaired self- and mother-referential processing capabilities found here.

Consistent with earlier findings^16,20,23^, during the encoding phase, behavioral data from this study demonstrated extended RTs across all experimental conditions for first-episode SZ patients relative to HC, confirming compromised processing speed in both early-stage and chronic SZ.

Thirdly, akin to the findings in first-episode SZ patients, this study reveals that individuals with BD also exhibit intact self-referential and mother-referential processing abilities, which contrasts with prior research^18^ suggesting impaired self-referential processing in BD relative to HC. Concurrently, earlier studies have indicated a negative correlation between self-referential bias scores and manic symptomatology in BD, meaning that more severe manic symptoms are associated with a greater degree of impairment in self-referential processing^18^.

In the present sample, BD participants were predominantly experiencing depressive episodes characterized by heightened depressive symptom severity (HAMD score: 20.67 ± 11.37 versus 6.2 ± 8.61), whereas past investigation^18^ involved subjects with more profound manic symptoms (YMRS score: 18.2 ± 10.53 compared to 8.00 ± 5.99). This difference in patient profiles may underlie the inconsistency between these two sets of findings. Moreover, previous literature^18^ has suggested that impairments in self-referential processing could be exclusive to BD cases accompanied by psychotic features. However, despite the presence of psychotic symptoms in our BD cohort as measured by a PANSS total score of 48.76 ± 8.80, no deficits in self-referential processing were detected. This implies that the relationship between self-referential processing capabilities and psychotic symptoms in BD might not be as strong as previously thought, and instead suggests that the phase of illness (i.e., manic or depressive episode) and the severity of related symptoms play a more substantial role. These observations underscore the need for further research to elucidate the complex relationship between BD's different phases, symptom severity, and self-referential processing functions. Future studies should delve deeper into this issue to clarify these dynamics.

Consistent with past literature^18^, BD patients displayed prolonged RTs across all experimental conditions during the encoding phase compared to HC, indicative of compromised information processing speed in this population.

Lastly, patients with MDD exhibited significantly superior recognition performance and corresponding bias indices for both self- and mother-referential processing when compared against those diagnosed with first-episode SZ and BD.

Notably, during the encoding phase of self-referential processing, individuals in the first-episode SZ group displayed longer RTs relative to their MDD counterparts. Furthermore, both first-episode SZ and BD patient groups demonstrated a more extended engagement duration when engaging in tasks requiring other-referential processing as compared to the MDD group. These findings suggest that at early stages of SZ and BD patients exhibit potentially reduced cognitive efficiency compared to MDD patients.

The clinical implications arising from this study are multifaceted. Initially, the present research enhances our comprehension of rumination symptoms by demonstrating that individuals with MDD exhibit a pronounced self-referential advantage effect, spending more time and engaging more deeply during encoding and retrieval of information pertaining to themselves. Secondly, this work, in conjunction with prior literature, underscores the heterogeneity among first-episode SZ patients regarding self-referential processing impairments. The variability observed may be linked to the severity of symptoms experienced by these patients. Consequently, assessing self-referential processing capacity could serve as a potential metric for gauging the effectiveness of pharmacological interventions in early-stage schizophrenia. Thirdly, while rumination has been recognized as a prevalent symptom across both BD's depressive and manic phases, as well as MDD^24,25^, our findings suggest distinct patterns of impairment in self-referential processing between these two psychiatric conditions. Notwithstanding the observation that BD participants in our study manifested more severe depressive symptoms than those in the depression group (mean scores: 20.67 ± 11.37 versus 15.57 ± 6.13), no impairments were detected in their self-referential processing abilities. The present study's findings, alongside previous research findings^18^, suggest that self-referential processing may serve as a potential adjunctive biomarker for differentiating between BD and MDD in diagnostic settings.

The present study has several limitations that warrant consideration. Firstly, the inherent cognitive disparities between patients and HC are substantiated by our findings, where both first-episode SZ and BD participants exhibited significantly prolonged RTs across all three experimental conditions during the encoding phase as compared to normal controls, indicative of compromised basic information processing in these patient populations. However, a notable distinction emerged during the recognition phase; while differential recognition scores were observed exclusively under self- and/or mother-related conditions for patients, no significant differences transpired in other-referential processing between the patient groups and normal controls. This observation suggests, to some extent, that the obtained results do not solely emanate from fundamental cognitive capacity discrepancies between patients and controls. In future research endeavors, it is crucial to incorporate a comprehensive cognitive assessment within the study design to more effectively control for potential confounding variables. Another limitation herein is the lack of consideration for the potential influence exerted by varying clinical symptomatology on the impairment patterns of self-referential processing in BD patients. Addressing this knowledge gap is essential for refining our understanding of the disorder-specific expressions of self-referential processing deficits. Lastly, the results of this study should be interpreted with caution, and future research necessitates the replication and validation of our findings in larger, more extensive cohorts.

In summary, the present study provides preliminary evidence for unique patterns of self-referential processing abilities in MDD, BD, and first-episode SZ. It emphasizes the potential different roles that self-referential processing may have in evaluating treatment response and aiding in discriminative diagnosis among these conditions.

## Material and methods

### The calculation of sample size

A power analysis was conducted using the G*Power software^26^ to ascertain the requisite sample sizes necessary for discerning significant differences between HC and patient cohorts with BD, first-episode SZ, and MDD. The principal outcome measure in these calculations was the recognition scores obtained from previously reported self-referential processing tasks^18,20,21^, which exhibited the following means and standard deviations: HC versus BD: 0.45 ± 0.03 vs. 0.26 ± 0.03; HC versus SZ: 0.47 ± 0.15 vs. 0.35 ± 0.18; and HC versus MDD: 0.36 ± 0.13 vs. 0.44 ± 0.13. With an alpha level set at α = 0.05 to maintain statistical significance, and a desired power (1 − β) of 0.90 to ensure adequate statistical power, the estimated minimum required sample sizes were determined as follows: 11 participants for the BD group, 33 participants for the first-episode SZ group, and 44 participants each for both the MDD group and the HC group.

### Participants

In the current study, guided by the aforesaid power analysis and sample size estimation standards, we recruited 41 patients with first-episode SZ, 33 individuals diagnosed with BD, and 44 participants suffering from MDD from Beijing HuiLongGuan Hospital. Concurrently, 44 HC were enrolled through local community advertisements.

Diagnoses of first-episode SZ, BD, and MDD were established according to the Diagnostic and Statistical Manual of Mental Disorders Fourth Edition (DSM-IV) criteria^27^, with each diagnosis confirmed by both an attending psychiatrist and subsequently verified by a senior psychiatrist.

For the first-episode SZ cohort, additional inclusion criteria included: (1) experiencing their inaugural episode of illness, operationally defined as either their initial treatment contact or an illness duration not exceeding three years since symptom onset; (2) being antipsychotic-naive, meaning no prior exposure to antipsychotics, or having minimal exposure quantified as a cumulative treatment period of ≤ 2 weeks. Regarding BD participants, they had to be in the acute phase or remission stage of a depressive episode. Exclusionary criteria applied specifically to those in a current manic episode or mixed affective state. Common exclusion criteria for both SZ and BD groups encompassed conditions such as overt brain injury, diagnosed neurological disorders, intellectual disability ascertained by an IQ score below 70, substance abuse or dependence within the last six months, and receipt of electroconvulsive therapy within the preceding six months. First-episode SZ and BD patients were drawn from the inpatient population. Enrollment was carried out after a well-defined period of relative clinical stability post-hospitalization, ensuring that SZ subjects displayed sufficient communicative competence and had comprehended the nature and implications of our experimental protocols to provide fully informed consent. This interval between hospital admission and study enrollment consistently averaged approximately 2–3 weeks.

For outpatient MDD participants, specific inclusion criteria required them to exhibit mild to moderate depression, evidenced by a Hamilton Depression Scale 17 items (HAMD-17) score of ≥ 7 but < 24 points^28^ . Exclusions for MDD patients included: (1) meeting DSM-IV axis I diagnostic criteria for any other mental disorder; (2) presenting with suicidal tendencies or scoring item 3 ≥ 3 on the relevant assessment tool; (3) showing psychotic symptoms or psychomotor retardation/hyperactivity symptoms.HC were free from DSM-IV Axis I disorders, had no history of substance abuse or dependence within the previous six months, and lacked first-degree relatives with a history of psychotic or affective disorders.

All participants were aged between 18 and 55 years, had completed at least 12 years of education, and possessed normal or corrected-to-normal visual acuity. Each participant voluntarily gave informed consent and received compensation for their participation. The entire research process strictly adhered to the ethical principles outlined in the Declaration of Helsinki (1964) and its subsequent revisions. Ethical clearance was granted by the Research Ethics Committee of Beijing HuiLongGuan Hospital on July 5th, 2016, under the assigned ethical approval number 2016–26.

In the overall study group, four MDD participants did not complete the full experimental procedure. Moreover, five MDD patients and three HC were excluded from statistical analyses due to their exceptionally high educational attainment that necessitated their removal during matching procedures to maintain homogeneity. Additionally, technical issues led to incomplete data for three HC, resulting in their exclusion. Therefore, the final dataset consisted of 41 first-episode SZ patients, 33 BD patients, 35 MDD patients, and 38 HC for subsequent statistical evaluations. No significant differences in age, education level, or sex distribution were detected across the four diagnostic categories. Clinical symptom ratings were carried out by a panel of four attending psychiatrists, achieving acceptable inter-rater reliability (kappa = 0.83). Symptom severity in SZ patients was evaluated using the Positive and Negative Syndrome Scale (PANSS)^29^, BD patients were assessed using both the Hamilton Depression Rating Scale (HAM-D) and the Young Mania Rating Scale (YMRS)^30^, while MDD patients were rated using the HAM-D and the Hamilton Anxiety Rating Scale (HAM-A)^31^. A comprehensive overview of the demographic and clinical characteristics of the study participants is presented in Table 3.

**Table 3:** Demographic and clinical data concerning patients with first-episode SZ, BD, MDD and for HC.

Characteristics	SZ (n = 41)	BD (n = 33)	MDD (n = 35)	HC (n = 38)	F/χ^2^	*p*
Mean age (years)	34.07 ± 6.87	34.03 ± 9.64	35.00 ± 7.99	34.5 ± 7.78	0.11	0.953
Education time (years)	15.60 ± 1.65	15.55 ± 1.70	16.23 ± 1.22	15.77 ± 2.39	1.05	0.373
Sex, male/female	14/27	13/20	12/23	16/22	0.75	0.862
Handedness, right/left	41/0	33/0	35/0	38/0	–	
PANSS total score	51.93 ± 25.64	48.76 ± 8.80	–	–		
Positive score	15.10 ± 6.84	11.30 ± 4.97	–	–		
Negative score	12.07 ± 7.11	10.09 ± 3.74	–	–		
General score	27.63 ± 9.37	27.45 ± 5.65	–	–		
Chlorpromazine equivalents^32^ (mg–day)	367.37 ± 219.62	–	–	–	–	
YMRS	–	8.00 ± 5.99	–	–	–	
HAM-A	–	–	19.89 ± 9.09	–	–	
HAM-D	–	20.67 ± 11.37	15.57 ± 6.13	–		

SZ: first-episode schizophrenia; BD: bipolar disorder; MDD: major depressive disorder; HC: healthy controls; PANSS: Positive and Negative Syndrome Scale; HAMD: Hamilton depression Scale; YMRS: Young Mania Rating Scale; HAM-A: Hamilton Anxiety Scale.

### The SRE task

**Figure 3:**
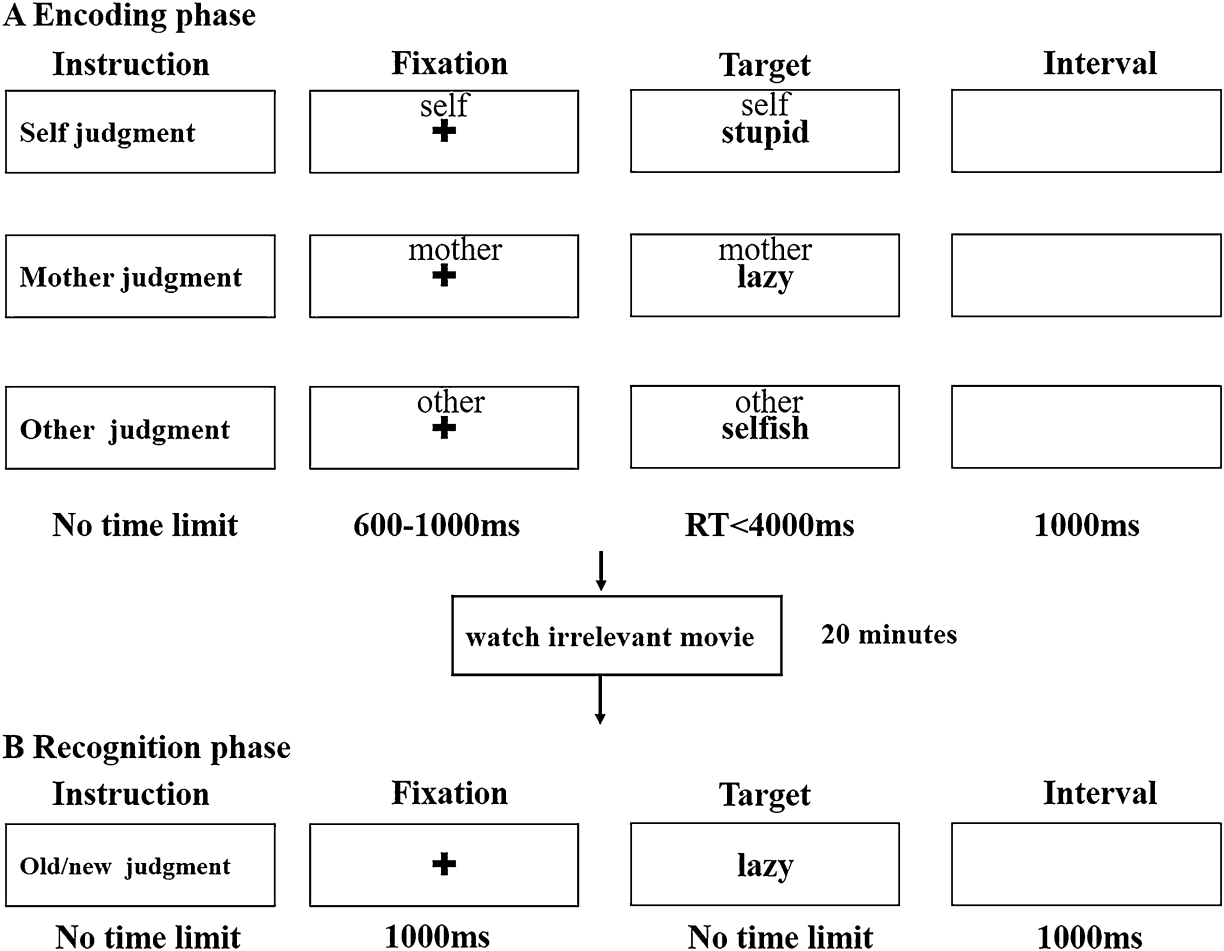
The general procedure of Self-referential effect task. An illustration of general procedure of self-referential effect task. Participants were instructed to finish encoding phase and recognition phase separated by irrelevant movie watching.

We used the SRE task previously described by our research group^20^ (Fig. 3). Briefly, the task consisted of two stages: an encoding phase and a recognition phase. In the encoding phase, the participants were asked to evaluate whether a certain personality-trait adjective presented in Chinese was appropriate to describe themselves, their mothers, and ‘other’, that is a well-known public figure (Hu Jintao, former President of the People's Republic of China). A total of 180 words were randomly selected for the encoding phase and there were 60 words in each condition (30 positive and 30 negative words). Each trial began with a gaze crossover of 600–1000 ms, followed by a “cue” word (self, mother, or other) above the adjective feature for a maximum of 4000 ms. Participants were required to respond as quickly and accurately as possible by pressing buttons on the response box with their left and right index fingers. The assignment of left and right button presses was counterbalanced across all participants. The target word disappeared when the participant indicated their response, and the next trial began 1000 ms later.

After the encoding session, the participants were asked to watch an unrelated video for 20 min, after which they completed an unexpected new/old word recognition test. All 180 words were included, along with 120 new characteristic adjectives. The participants were required to determine whether certain words had been present during the encoding phase.

### Behavioral measures

E-Prime software (Version 3.0, Psychology Software Tools, Inc., Pittsburgh, PA, USA) was used for stimulus display and data acquisition. Data analyses were conducted using SPSS Statistics 20.0 (IBM, USA).

During the encoding phase, apart from the average RT per task condition, we calculated the differential RT by subtracting the RT under the other-referential condition from the corresponding RT in the self-referential (yielding SR bias RT) and mother-referential conditions (yielding MR bias RT), as well as the difference between the self-referential and mother-referential RT (designated as SM bias RT).

During the recognition phase, we adhered to signal detection theory as previously established^4,7,9^ to operationalize two pivotal indices: the first being the sensitivity index d', which is computed as the difference between the hit rate and false alarm rate; a higher d' signifies superior recognition performance, indicating an enhanced ability to discriminate signals from noise. The second critical variable was the response bias or criterion value. In the present investigation, we derived three unique response bias scores based on these calculations: the SRE bias score, the Mother-Referential Effect (MRE) bias score, and the Self-Mother (SM) bias score. These were respectively defined as the difference between the “self-referential d” and the “other-referential d” (“self-referential d” minus “other-referential d”), the difference between the “mother-referential d” and the “other-referential d” (“mother-referential d” minus “other-referential d”), and the difference between the “self-referential d” and the “mother-referential d” (“self-referential d” minus “mother-referential d”).

### Statistics

In the context of behavioral data collected during the encoding phase, a 4 (group: HC, MDD, BD, and first-episode SZ) × 3 (encoding condition: self-referential, mother-referential, and other-referential) repeated-measures analysis of variance (ANOVA) was employed to meticulously examine RT. The independent between-subjects factor reflected group membership, while the within-subjects factor denoted the different encoding contexts. This rigorous analytical approach was specifically tailored to discern potential interaction effects between diagnostic groups and various encoding conditions.

Furthermore, a separate 4 × 3 repeated measures ANOVA was conducted to scrutinize RT biases corresponding SR bias RT, MR bias RT, and SM bias RT. This investigation aimed at revealing any diagnostic category-specific variations in the distinct patterns of bias that emerged during the encoding process.

During the subsequent recognition phase, analogous repeated measures ANOVAs with an identical design as used in the encoding stage were applied independently to recognition scores and bias scores. Throughout this study, statistical significance was set at *α* = 0.05. Whenever significant interactions were detected, post-hoc analyses were conducted using simple effects models, accompanied by appropriate adjustments for multiple comparisons to mitigate family-wise error rate.

## Data Availability

The raw data and/or analyzed datasets of the present study will be made available by the corresponding author on reasonable request.
